# The Aerial Parts of *Bupleurum Chinense* DC. Aromatic Oil Attenuate Kainic Acid-Induced Epilepsy-Like Behavior and Its Potential Mechanisms

**DOI:** 10.1155/2022/1234612

**Published:** 2022-04-11

**Authors:** Xiaomao Li, Yan Liu, Siyi Wang, Yikai Jiang, Adnan Mohammed Algradi, Yuanyuan Zhou, Juan Pan, Wei Guan, Haixue Kuang, Bingyou Yang

**Affiliations:** Key Laboratory of Basic and Application Research of Beiyao (Heilongjiang University of Chinese Medicine), Ministry of Education, Heilongjiang University of Chinese Medicine, 24 Heping Road, Xiangfang District, Harbin 150040, China

## Abstract

The aerial parts of *Bupleurum Chinense* DC. aromatic oil (BAO) were a well-known Chinese herbal medicine plant extract used to treat epilepsy. This study aimed to explore the therapeutic effect of BAO on kainic acid- (KA-) induced epileptic rats and the possible mechanism of its antiepileptic effect. The composition and content of BAO were analyzed by GC-MS, and BAO was administered orally to alleviate the epileptic behavior induced by KA brain injection. The behavior of epileptic rats was determined by Racine grading criteria. And hematoxylin-eosin staining (HE), Nissl staining, immunohistochemistry, Elisa, Western blot, and other methods were used to study the antiepileptic mechanism of BAO, and the possible mechanism was verified by the epileptic cell model of hippocampal neurons induced by the low-Mg^2+^ extracellular fluid. BAO was mainly composed of terpenoids and aliphatic compounds. And BAO could improve KA-induced epilepsy-like behavior, neuroinflammation, and neurotransmitter abnormalities in the hippocampus. Furthermore, BAO could regulate the expression of GABA, NMDAR1, Notch1, and MAP2 to improve the symptoms of epilepsy. These results were also validated at the cellular level. These results indicated that BAO could alleviate the epilepsy-like behavior through the action of the Notch/NMDAR/GABA pathway.

## 1. Introduction

Statistical data showed that the prevalence rate of epilepsy in China was 7.0‰. According to this estimate, there were about 9 million epilepsy patients in China, of which about 25% were refractory epilepsy, such patients could not effectively control their seizures with drugs. Among them, temporal lobe epilepsy (TLE) patients account for about 70% [1]. Although modern antiepileptic drugs (AEDs) could control about 82% of patients with seizures, there were still 1/5 patients with recurrent seizures, and because patients with epilepsy need long-term or even lifelong medication treatment, the toxic side effects of chemosynthetic drugs were particularly prominent [2]. The existing first-line AEDs almost had potentially adverse effects on the behavior and cognitive function of patients with epilepsy. Therefore, it was particularly important to explore the prevention and treatment ideas and find safer and more efficient therapies and drugs with less toxic side effects and definite curative effects. At this time, Chinese herbal medicine had attracted much attention because of its unique curative effect.

The occurrence of epilepsy was the result of the imbalance of excitation-inhibition in the brain. As the most representative inhibitory neurotransmitter in the central nervous system, the change of GABA extracellular concentration played a pivotal role in the process of neuron excitability change and abnormal discharge. However, as a rate-limiting enzyme of GABA synthesis, glutamate decarboxylase (GAD) had been paid more and more attention to its relationship with epilepsy [3].

*B. chinense*, belonging to the plant family *Bupleurum* spp., was a well-known traditional Chinese medicine (TCM) that had been used for more than a thousand years, distributed mainly in Heilongjiang, Liaoning, Jilin, and Neimeng province of China [4]. It was reported that the *B. chinense* and their aerial parts had similar chemical components and pharmacological effects have a good therapeutic effect on epilepsy. In addition, some studies showed that the aerial parts of *B. chinense* had an obvious antiepileptic effect, and the curative effect was better than that of *B. chinense* [5]. Some kinds of literature have revealed that a variety of volatile oils could protect and repair GABA neurons, improve the synthesis and extracellular release of GABA transmitters in GABA neurons, reduce the reabsorption and degradation of GABA, increase the GABA content in the whole brain tissue, reduce the excitability, and reduce their excitotoxicity to nerve tissue through inhibitory postsynaptic potential, to inhibit the abnormal discharge of epilepsy and play an antiepileptic role [6].

Therefore, this study aimed to explore the effect of the aerial parts of *B. chinense* aromatic oil (BAO) on KA-induced epileptic behavior in rats through GABA regulation.

## 2. Materials and Methods

### 2.1. Chemicals

Kainic acid was obtained from Sigma-Aldrich (Shanghai, China); sodium valproate oral solution was obtained from Sanofi (Hangzhou, China); and Dian Xian Ning tablet was purchased from Kunming Chinese Medicine Factory Co. Ltd. (Kunming, China). And all other analytical reagents were purchased from Sigma-Aldrich (Shanghai, China).

### 2.2. Plant Extract

The aerial parts of *B. chinense* were collected in Daqing, Heilongjiang Province, China, in 2017 and identified as *Bupleurum Chinese* DC by Professor Rui-Feng Fan from Heilongjiang University of Traditional Chinese Medicine. The aerial parts were stored in the laboratory of Chinese Medicine Chemistry of the Heilongjiang University of Chinese Medicine with specimen number 20170910.

BAO was obtained by steam distillation. The BAO sample was diluted with anhydrous ether, dried with a little anhydrous sodium sulfate and identified by GC-MS (Agilent, USA). The gas chromatographic column was a quartz capillary column (30 m ×0.25 mm ×0.25 *μ*m) with N_2_ carrier gas. The column flow rate was 1 mL/min; the shunt ratio was 20: 1. The inlet temperature was 300°C, and the temperature rise rate was 60°C/min, 10°C/min to 160°C, 2°C/min to 210°C, 10°C/min to 300°C. For mass spectrometry, the carrier gas was He, ionization mode is EI, electron energy is 70 eV, ion source temperature is 20°C, scanning speed was 1 s, and scanning range is 33~550 m/z. Anhydrous ether was diluted and injected at 0.6 *μ*L.

### 2.3. Animals

70 specific pathogen-free male Sprague-Dawley rats, weighing 180~200 g, were purchased from Liaoning Changsheng Biotechnology Co., Ltd. (SCXK (Liao) 2020-0001). Experimental animals were raised under standardized conditions, the temperature was 20~23°C and the humidity was 50% ~60%. All experiments were approved by the Animal Experiment Committee of Heilongjiang University of Chinese Medicine (20201230001).

### 2.4. Primary Cell Culture

Rat hippocampal neurons from 1 to 3 days old SD rats were cultured in neuronal culture medium containing 98% neurobasal medium (Gibco, Carlsbad, CA), 2% B27 supplement (Gibco, Carlsbad, CA) at 37°C under 5% CO_2_. The low-Mg^2+^ extracellular fluid (ECF) was configured according to previous methods [7]. The neurons were cultured in the low-Mg2+ ECF (2 mL per well) at 37°C under 5% CO2. After 3 h, the neurons were rinsed twice with a neuronal culture medium and then cultured in a neuronal culture medium with VAP (1 mM) and BAO (50, 100, 200 *μ*g/mL) at 2 mL per well. After 24 h, the cell viability was evaluated by CCK-8 kit assay [7].

### 2.5. Experimental Design

After 60 rats were anesthetized, the lateral ventricle was stereo-positioned, and a custom-made microinjection tube was used to lower a needle of 3.8 mm into the right lateral ventricle with a uniform injection of 3 *μ* of KA solution at a concentration of 0.5 *μ*g/*μ*L (50 rats) or 3 *μ* normal saline (10 rats). After the injection, the needle was retained for 5 min to establish an animal model of epilepsy caused by KA [8].

50 successful male SD rats were randomly divided into 5 groups with 10 rats in each group, model group, western medicine positive drug group (sodium valproate oral VAP, 180 mg/kg, b.w., po.), traditional Chinese medicine positive drug (Dianxianning tablets, DXN, 500 mg/kg, b.w., po.), BAO high-dose group (BAOH, 200 mg/kg, b.w., po.), and BAO low-dose group (BAOL, 50 mg/kg, b.w., po.).

### 2.6. Immunohistochemistry

Brain slices were immersed in 0.1 mol/L phosphate buffer (PBS) containing 0.5% Triton X-100 for 60 min and 0.6% H_2_O_2_ ethanol saline solution (50% ethanol and 0.9% sodium chloride) for 45 min. Normal sheep serum was incubated for 60 min, poured out without washing, and dripping with rabbit-derived glial fibrillary acidic protein (GFAP) polyclonal antibody (Abcolonal, Wuhan, China, 1 : 500), and incubated at 4°C for 36~48 h. Biotinylated secondary antibody (sheep antirabbit) was added dropwise and incubated at room temperature for 2 h. Streptavidin labeled with horseradish enzyme was added dropwise and incubated at room temperature for 2 h. Between each step, it was washed with 0.01 mol/L PBS 3 times for 10 m each time. After that, it was colored with 3,3'-diaminobenzidine tetrahydrochloride (DAB), counterstained with hematoxylin, dehydrated, transparent, and sealed. The fluorescence intensity of the hippocampus was counted at 200× magnification.

### 2.7. Biochemical Analysis

Total protein was extracted from fresh hippocampal tissues of experimental rats in each group, and caspase3, COX2, TLR4, HMBG1, NMDAR1, GABA AR, GAD65, GAD67, GAFP, and GIRK1 expression were determined. For specific methods, please refer to the instructions of Shanghai Enzyme-linked Biotechnology Co., Ltd. ELISA kits.

### 2.8. Western Blot Analysis

Total protein was extracted from fresh and interfered hippocampal tissues of rats in each group. The total protein was separated by polyacrylamide gel electrophoresis with a 20 *μ*g sample amount and then transformed into the membrane. After sealing, the protein was incubated with primary antibody Bcl2, Bax, caspase 3, GAD65, GAD67, GIRK1, GAFP, GABA AR, Notch1, MAP2, and NMDAR1 (1 : 1000, Abcolonal, Wuhan, China) at 4°C overnight. The ECL reagent was incubated at room temperature for 1 h with an appropriate dilution concentration of corresponding HRP-labeled secondary antibody (Abcolonal, Wuhan, China). Take GADPH (1 : 1000, Abcolonal, Wuhan, China) as the internal reference. The results were analyzed with the expression level of each control group as the standard and compared with other groups.

### 2.9. Statistical Analysis

SPSS17.0 statistical software (SPSS Inc., Chicago, IL, USA) was used to analyze the data. The data of the experimental results were expressed by mean ± standard error (X ± SEM), and the data were analyzed by one-way ANOVA. The homogeneity test of variance was performed first. After the test of variance, the difference between the groups was statistically significant. LSD-test was used to compare the difference, and the difference was statistically significant when *P* ≤ 0.05.

## 3. Results

### 3.1. The Chemical Components of BAO

Compared with the NIST Spectral Library, the aromatic oil is isolated from the aerial parts of *B. chinense* which resulted in the identification of 63 different components, mainly containing terpenoids compounds, detailed in Table 1, and the GC chromatogram of BAO and MS of all major identified components in Supplemental Files Figures S1 and S2.

### 3.2. BAO Attenuated KA-Induced Epilepsy-like Behavior

**Figure 1:**
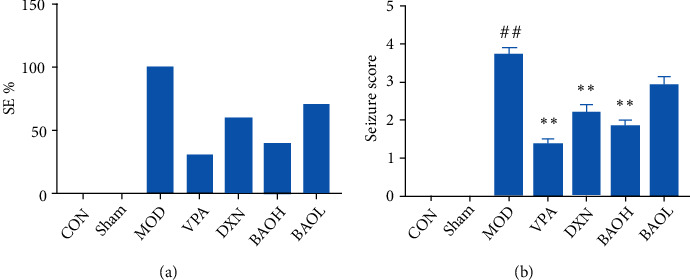
Effect on improving the symptom of SE in rats with epilepsy KA-induced. (a) SE% of epilepsy rats after treatment. (b) Seizure core of epilepsy rats after treatment. Compared with CON and sham groups, the difference was significant (#*P* < 0.05). Compared with the MOD group, the difference was significant (∗*P* < 0.05).

Compared with the control group (CON) rats, the model group (MOD) rats developed IV-V convulsive generalized tonic-clonic seizures, forelimb spasms upright, and falls; compared with the model group, the positive drug group (VPA) could significantly improve the symptoms of epilepsy in rats and can significantly prolong the incubation period and duration of convulsions in rats. Compared with the model group, BAOH could improve the symptoms of epilepsy in rats, reduce the incidence of SE% (*P* < 0.05) (Figure 1), and prolong the incubation period and duration of convulsions in model rats. But BAOL did not improve significantly.

### 3.3. Observation of He Staining and Nissl Staining

**Figure 2:**
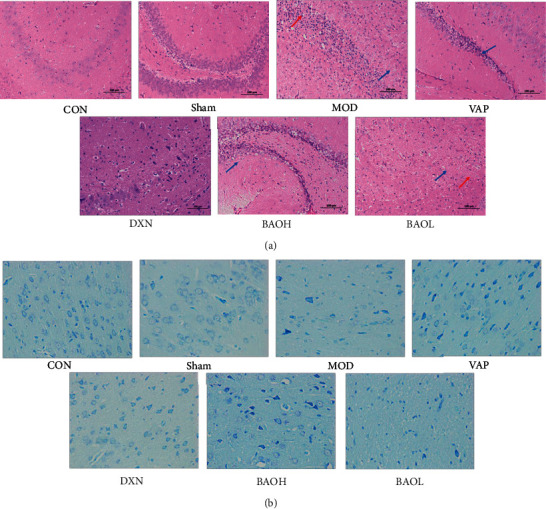
Effect on the pathological changes in KA-induced epilepsy rats with treatment. (a) HE staining in the hippocampus.. (b) Nissl staining in the hippocampus.

Under a light microscope, it was found that BAO could reduce the histological damage caused by KA. Compared with the sham operation group, KA could significantly change the morphology of hippocampal CA3 area of brain tissue: loss of pyramidal cells and Nissl bodies, pyknosis of nuclei, hypertrophy, and massive proliferation of astrocytes and disordered and irregular arrangement of pyramidal cell layers. The pathological changes and neuron loss in the hippocampal CA3 area of brain tissue in the BAOH treatment group are significantly reduced, as shown in Figure 2.

### 3.4. Observation of Immunohistochemistry

**Figure 3:**
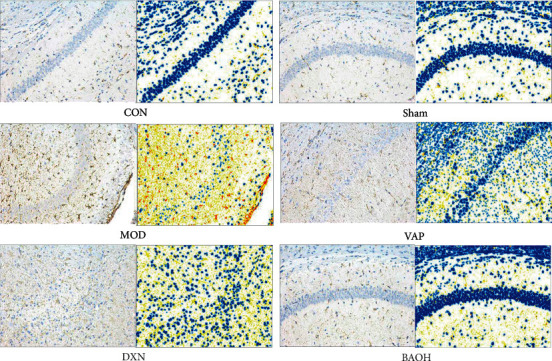
Effect on the expression of GFAP in KA-induced epilepsy rats with treatment.

In the CON group and sham group, there were fewer GFAP immunoreactive cells with thin cell bodies and protruding fibers, mainly located outside pyramidal cells and granulosa cell layers. After KA induction, edema appeared, which was manifested as hypertrophy, deep staining, deformation, and disarrangement of the cell body, and the GFAP-positive area increased significantly. However, BAOH could ameliorate the abnormal GFAP-positive area induced by KA (*P* < 0.01; Figure 3). These results suggested that BAO could inhibit hippocampal neuron loss by activating astrocytes, which was consistent with morphological changes.

### 3.5. Changes in Biochemical Indicators

A large number of previous studies have confirmed that the expression of caspase 3 increases after SE and caspase 3 inhibitors could reduce neuronal apoptosis after SE [9]. caspase 3 was a key protease in the caspase family. It had the characteristic of specifically cutting chromosomal DNA after aspartic acid, which ultimately led to the phasic degradation of DNA [10]. Therefore, caspase 3 was also called killer protease. These results showed a similar conclusion; KA could improve the expression of caspase 3, but treatment with BAO could reduce the caspase 3 expression.

After the seizure, neurons, astrocytes, and microglia quickly released damage-associated molecular patterns (DAMPs). At the same time, neurons and astrocytes had toll-like receptors (toll-like receptors, TLRs) activation, which was considered to be an important process of neuroinflammation caused by epileptic seizures [11]. Through Elisa's analysis, BAO could improve inflammatory protein abnormalities.

**Figure 4:**
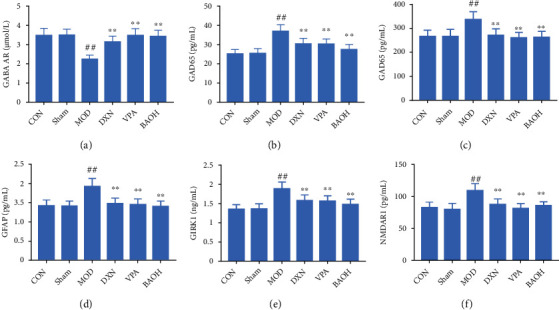
Effect on the changes of neurotransmitter levels in KA-induced epilepsy rats with treatment. (a) The content of GABA AR in KA-induced epilepsy rats with treatment; (b) the content of GAD65 in KA-induced epilepsy rats with treatment; (c) the content of GAD67 in KA-induced epilepsy rats with treatment; (d) the content of GFAP in KA-induced epilepsy rats with treatment; (e) the content of GIRK1 in KA-induced epilepsy rats with treatment; and (f) the content of NMDAR1 in KA-induced epilepsy rats with treatment.

At present, neurotransmitters had been known to play an important role in the pathogenesis of epilepsy pain; that was, the excitation and inhibition process in the brain was mediated by various neurotransmitters, GABA and glutamate were the most representative transmitters involved in inhibition and excitation, and their metabolic abnormalities were closely related to the occurrence of epilepsy, while NMDAR was the ionic receptor of Glu [12, 13]. At the same time, studies have shown that there was a GAD65-GABA-GABA AR-G protein-GIRK pathway between synapses. GABA was synthesized by glutamate under the catalytic action of GAD65, released into the synaptic cleft, and bound to the N-terminal of the GABAB receptor which was fused with G protein in the postsynaptic membrane to activate the GABAB receptor [14]. After the activation of the GABAB receptor bound to the corresponding G protein, the GIRK channel opened, K^+^ influx increases, and the postsynaptic membrane hyperpolarizes, resulting in postsynaptic inhibition and inhibiting the proliferation of neuronal hyperexcitability. This pathway suggested that GAD65 was related to the effect of GIRK 1 [15]. Our results showed that BAO could improve the inhibitory GABA neurotransmission abnormalities (Figure 4).

### 3.6. BAO through GABA Regulation Attenuated KA-Induced Epilepsy-like Behavior

The neuronal hyperexcitability caused by the combination of KA and its receptor might be the main cause of neuronal death. KA could decrease the expression of Bcl2 and increase the expressions of Bax and caspase 3. Meanwhile, BAO could reduce neuronal cell apoptosis by improving Bcl2/Bax/caspase 3 pathway.

At present, it was known that neurotransmitters play an important role in the pathogenesis of epilepsy and pain; that was, the process of excitation and inhibition in the brain is mediated by various neurotransmitters. KA could increase the expressions of GAD65, GAD67, GIRK1, and GFAP and decrease GABA AR expression. Meanwhile, BAO could regulate the GABA signal through GAD/GIRK1 pathway.

Notch 1 knockdown increased the expression of GAD67 in the hippocampus. GAD67 was one of several forms of glutamate decarboxylase, which was responsible for catalyzing glutamate to produce GABA [16]. And Notch could influence NMDAR functions [17]. We could find that BAO could regulate Notch1 through MAP 2 and NMDAR 1 to attenuate the KA-induced epilepsy-like behavior.

### 3.7. The Effect of BAO on Primary Neuronal Cells

**Figure 5:**
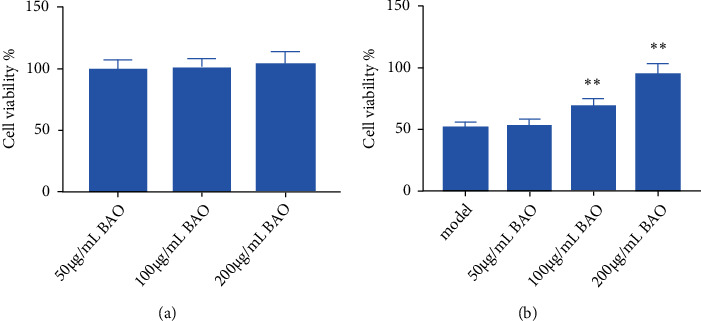
The effect of BAO on hippocampal neuron model of epilepsy. (a) The cell viability of different doses of BAO. (b) The effect of different doses of BAO on hippocampal neuron model of epilepsy. Compared with the model group, the difference was significant (∗*P* < 0.05).

After the addition of low-Mg^2+^ ECF, the cell viability of hippocampal neurons was significantly decreased (*P* < 0.01), indicating the presence of neuron damage in the low-Mg^2+^ ECF-induced refractory epileptic cell model. The cell viability of neurons in BAO concentration groups is significantly increased (*P* < 0.01), and there is a dose-effect relationship, and 200 *μ*g/mL had the most obvious effect, as shown in Figure 5. Results showed that BAO could improve neuronal apoptosis.

### 3.8. BAO through GABA Regulation Attenuated Hippocampal Neuron Model of Epilepsy

In refractory epilepsy, repeated and prolonged seizures tend to result in selective brain damage, including neuronal necrosis and apoptosis, mainly in the hippocampus. The loss and death of neurons were also observed in the low-Mg^2+^ ECF epileptic cell model. And BAO could regulate the abnormal expression of Bcl2, Bax, and caspase 3.

When the survival environment of neurons was changed, the neurons cultured in vitro could be induced to produce electrical seizures, which was similar to the abnormal electrical activity of neurons in human epilepsy. Mg^2+^ played a very important role in maintaining normal brain function. Studies have shown that Mg^2+^ was an important regulator of NMDAR mediated ion flow, which played a key role in excitatory nerve injury [18]. And Notch1 knockdown increased the expression of GAD67 in the hippocampus. GAD67 was one of several forms of glutamate decarboxylase, which was responsible for catalyzing glutamate to produce GABA [16, 17]. Our results showed that the expressions of Notch 1, MAP 2, NMDAR1, GAD65, GAD 67, and GIRK1 were increased; the expression of GABA AR was decreased in the Mg^2+^-free-treated neurons, while BAO could improve the abnormal discharge of neurons by regulating Notch1/GABA/GAD/GIRK.

## 4. Discussion

The aerial parts of *B. Chinense* aromatic oil (BAO) were a well-known Chinese herbal medicine plant extract used to treat epilepsy, and we found that BAO was rich in terpenoids. An increasing body of scientific literature indicates that terpenoids have multiple pharmaceutical functions, including antiepileptic activities [19, 20]. (+)-Borneol, a bicyclic monoterpene, is a common component of essential oils of medicinal herbs. This monoterpene is a positive modulator of human recombinant *α*1*β*2*γ*2L GABAA receptors at low concentrations of GABA [21]. Quintans-Júnior, Guimarães [22] revealed that pretreatment with (-)-borneol, carvacrol (phenolic monoterpene), and citral exerted a protective effect in PTZ-and MES-induced convulsions in mice. (-) Borneol and citral were more effective than carvacrol for all doses (50, 100, 200 mg/kg, i.p), resulting in increased time onset of clonic convulsions, while carvacrol was effective only at the highest dose [22]. Antagonism of PTZ-induced convulsions suggests that the terpenoids may have effects on GABAergic neurotransmission. According to NIST Spectral Library identification and analysis, BAO contains all of the above antiepileptic ingredients. Therefore, we hypothesized that BAO might ameliorate the effects of epilepsy by regulating GABA signaling pathway.

**Figure 6:**
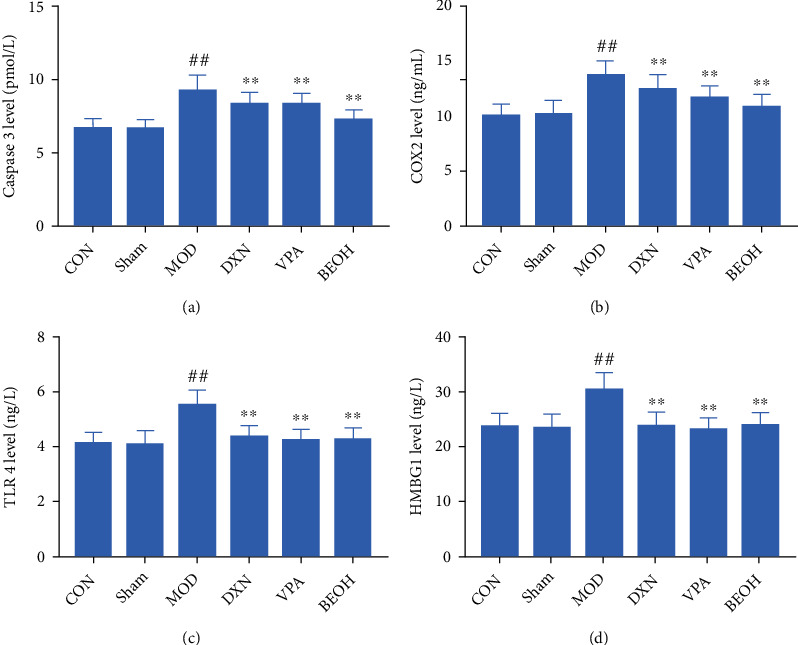
Effect on the expression of apoptosis and inflammatory proteins in KA-induced epilepsy rats with treatment. (a) The content of caspase 3 in KA-induced epilepsy rats with treatment; (b) the content of COX2 in KA-induced epilepsy rats with treatment; (c) the content of TLR 4 in KA-induced epilepsy rats with treatment; and (d) the content of HMBG1 in KA-induced epilepsy rats with treatment.

**Figure 7:**
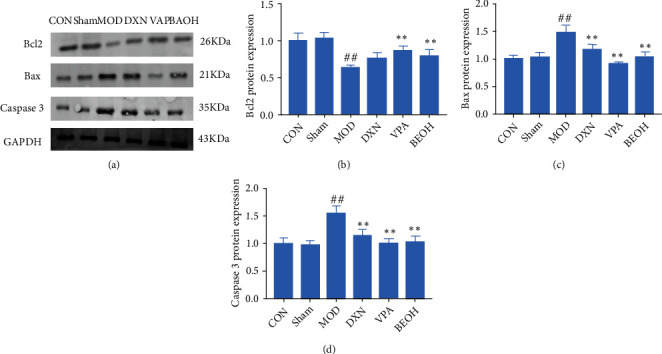
BAO through apoptosis pathway regulation attenuated KA-induced epilepsy-like behavior. (a) Western blotting in KA-induced epilepsy rats with treatment; (b) the expression of Bcl2 in KA-induced epilepsy rats with treatment; (c) the expression of Bax in KA-induced epilepsy rats with treatment; and (d) the expression of caspase 3 in KA-induced epilepsy rats with treatment.

**Figure 8:**
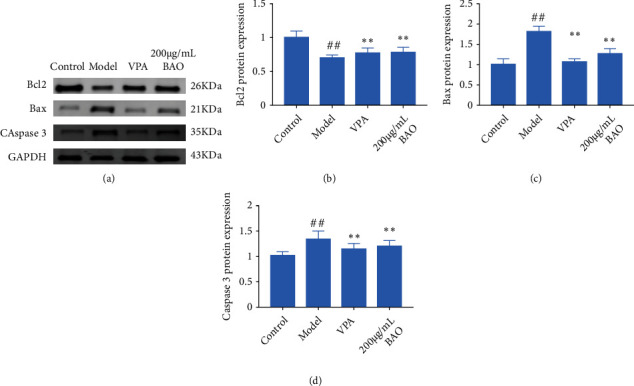
BAO through apoptosis pathway regulation attenuated the hippocampal neuron model of epilepsy. (a) Western blotting in hippocampal neuron model of epilepsy with treatment; (b) the expression of Bcl2 in hippocampal neuron model of epilepsy with treatment; (c) the expression of Bax in hippocampal neuron model of epilepsy with treatment; and (d) the expression of caspase 3 in hippocampal neuron model of epilepsy rats with treatment.

Based on BAO's ability to improve epileptic behavior and hippocampal injury, the mechanism of BAO's epileptic treatment was explored (Figures 1–4, 6). In human temporal lobe epilepsy patients, as well as in various epileptic models such as electric or chemical lighting and local or systematic administration of epileptic agents, post-epileptic neuronal death was typically characterized by apoptosis. At the same time, the Bcl-2 gene family played an important role in the process of cell apoptosis, and caspase 3 was a key protease in mammalian cell apoptosis, located at the core of the apoptotic cascade pathway. Once caspase 3 was activated, cell apoptosis was inevitable [23, 24]. The results showed that the expression levels of caspase 3 and Bax protein in the hippocampus of KA epileptic rats were significantly higher than those of CON and sham groups, while the expression levels of Bcl-2 protein were opposite (Figures 7 and 8), while BAO could inhibit the apoptosis of epileptic neurons by interfering with the abnormal expression of caspase 3, Bax, and Bcl-2 proteins.

**Figure 9:**
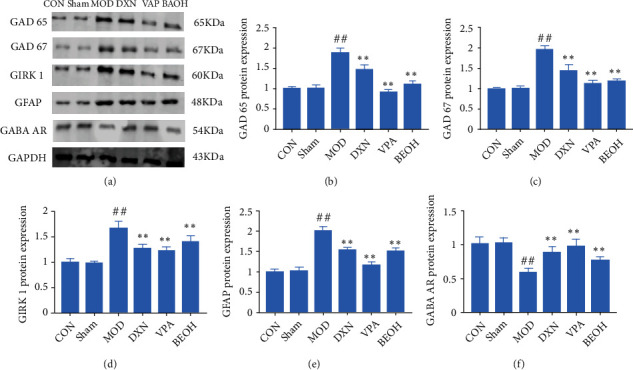
BAO through GABA pathway regulation attenuated KA-induced epilepsy-like behavior. (a) Western blotting in KA-induced epilepsy rats with treatment; (b) the expression ofGAD65 in KA-induced epilepsy rats with treatment; (c) the expression of GAD67 in KA-induced epilepsy rats with treatment; (d) the expression of GIRK1 in KA-induced epilepsy rats with treatment; (e) the expression of GFAP in KA-induced epilepsy rats with treatment; and (f) the expression of GABA AR in KA-induced epilepsy rats with treatment.

**Figure 10:**
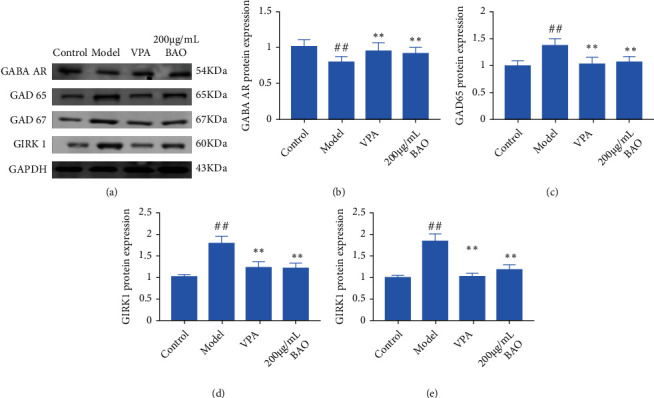
BAO through GABA pathway regulation attenuated the hippocampal neuron model of epilepsy. (a) Western blotting in hippocampal neuron model of epilepsy with treatment; (b) the expression of GABA AR in hippocampal neuron model of epilepsy with treatment; (c) the expression of GAD 65 in hippocampal neuron model of epilepsy with treatment; (d) the expression of GAD67 in hippocampal neuron model of epilepsy rats with treatment; and (f) the expression of GIRK1 in hippocampal neuron model of epilepsy rats with treatment.

Astrocytes were involved in many important physiological and pathological processes in the central nervous system, including epilepsy. GFAP was specifically expressed in astrocytes. It was a characteristic marker of astrocytes. The increased expression of GFAP and the changes in astrocyte morphology suggested astrocyte activation. On the one hand, activated astrocytes protect neurons by secreting various factors; on the other hand, due to the loss of their function of regulating K^+^ homeostasis, abnormal neuronal excitation and recurrent seizures would be caused by activated astrocytes. GIRK also played an important role in maintaining cell membrane resting potential and K^+^ internal environment stability [25, 26]. In this study, it was found that GFAP-positive expression area and GIRK1 protein expression increased after an epileptic seizure, while GFAP and GIRK1 expression decreased significantly after BAO treatment (*P* < 0.01). These results suggested that BAO might partially inhibit the expression of GFAP, weaken the activation of astrocytes, and regulate K+ homeostasis in epileptic seizures (Figures 9 and 10).

**Figure 11:**
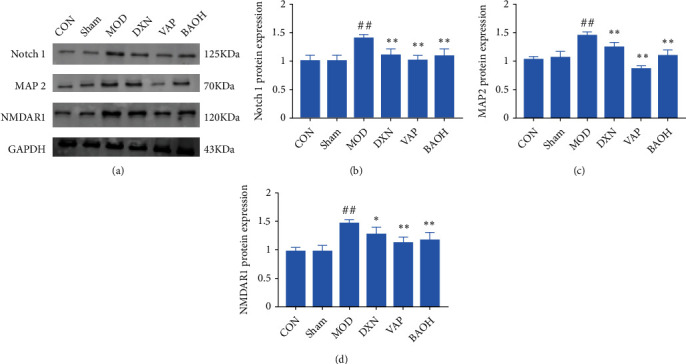
BAO through Notch/NMDAR pathway regulation attenuated KA-induced epilepsy-like behavior. (a) Western blotting in KA-induced epilepsy rats with treatment; (b) the expression of Notch1 in KA-induced epilepsy rats with treatment; (c) the expression of MAP2 in KA-induced epilepsy rats with treatment; and (d) the expression of NMDAR1 in KA-induced epilepsy rats with treatment.

**Figure 12:**
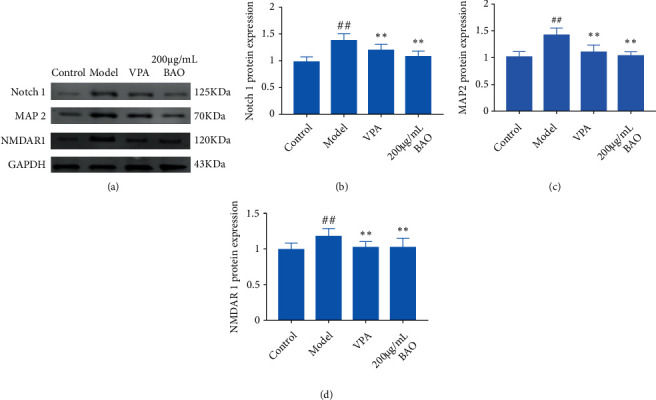
BAO through Notch/NMDAR pathway regulation attenuated hippocampal neuron model of epilepsy. (a) Western blotting in hippocampal neuron model of epilepsy with treatment; (b) the expression of Notch 1 in hippocampal neuron model of epilepsy with treatment; (c) the expression of MAP 2 in hippocampal neuron model of epilepsy with treatment; (d) the expression of NMDAR1 in hippocampal neuron model of epilepsy rats with treatment.

Notch signaling played an important role in TLE neuroinflammation and neuron injury. A large number of studies have shown that Notch signaling was activated in both TLE model rats and people with TLE [27]. Consistent with previous studies, we found that Notch-1 and its downstream target gene MAP2 were significantly higher in the MOD group than in the CON and sham groups, and BAO could down-regulate Notch and MAP2 expression (Figures 11 and 12). Thus, we could speculate that Notch can affect MAP2 expression by regulating TLRs.

**Figure 13:**
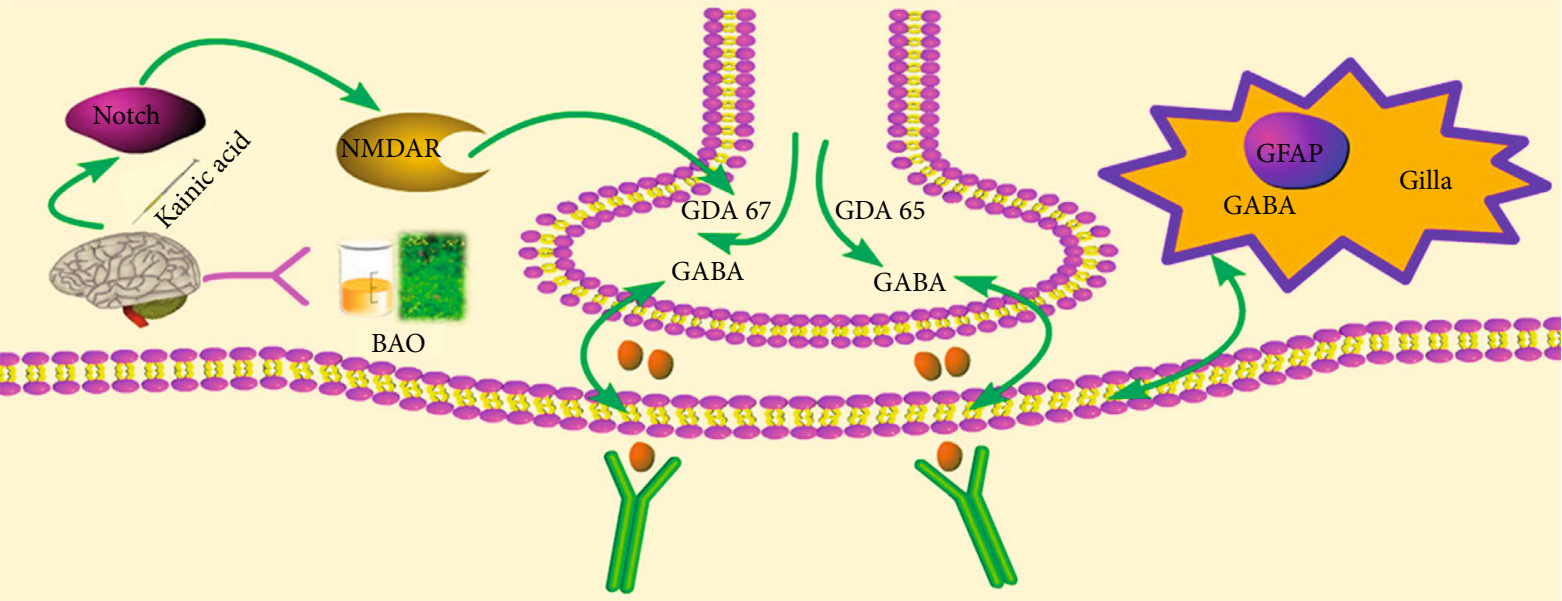
The mechanisms of the aerial parts of *B. chinense* aromatic oil attenuate kainic acid-induced epilepsy-like behavior.

There was a lot of evidence that NMDAR was involved in the pathophysiology of some mental diseases, such as schizophrenia, bipolar depression, and drug addiction [28, 29]. Previous studies had found that Notch1 expression was reduced in chronic unpredictable mild stress mouse models. Interestingly, nicotine significantly increased the expression of this protein, indicating a close relationship between NMDAR and the Notch1 receptor [30]. In addition, in the past decade, NMDAR antagonists had been used to establish schizophrenia models [31, 32], and their compounds were suitable for the treatment of bipolar depression [33]. Abnormal protein expression in neurological and psychiatric diseases was related not only to excitatory proteins but also to inhibitory proteins. For example, abnormal frontal GABA function in patients with schizophrenia was significantly associated with gamma oscillation [34]. From the perspective of neuronal oscillation, it was generally believed that the generation and modulation of theta and gamma oscillations are closely related to glutamate and GABAergic neurons [35, 36]. The knockdown of Notch1 increased the expression of GAD67 in the hippocampus, one of several forms of glutamate decarboxylase, which was responsible for catalyzing glutamate to produce GABA [16, 17]. Our results showed that BAO could regulate the Notch/NMDAR/GABA pathway to attenuate kainic acid-induced epilepsy-like behavior (Figure 13).

This study has been clear about the BAO antiepileptic active ingredients but did not reveal what compounds are significantly active ingredients in the treatment of epilepsy. The antiepileptic activities of various terpenoids from BAO will be further investigated in the following studies; researching and developing activity significantly on natural products lay the foundation for the treatment of epilepsy, at the same time provide a basis for the research of antiepileptic drugs.

## 5. Conclusions

In conclusion, the main active components of BAO are terpenoids, which can alleviate KA-induced epileptic behavior and play a therapeutic role in epilepsy by regulating Notch/NMDAR/GABA signaling pathway.

## Figures and Tables

**Table 1:** The chemical components of BAO by GC-MS.

No.	tR (min)	Name	Molecular formulae	Molecular weight g/Mol	Fixed area Proportion (%)
1	14.03	(H) Pyrrole-2-carboxaldehyde	C_5_H_5_NO	95.10	23.17
2	14.58	(H) Camphene	C_10_H_16_	136.23	2.36
3	15.39	(H) Bicyclol [3.1.0] hex-2-ene	C_10_H_16_	136.23	2.35
4	15.55	(H) *π*pinene	C_10_H_16_	136.23	13.52
5	15.87	2-pentylfuran	C_9_H_14_O	138.21	6.15
6	17.09	1-Methyl-2(-1-methyl)-benzene	C_9_H_11_	119.19	44.34
7	17.26	(R) D-limonene	C_10_H_16_	136.23	31.72
8	19.45	(H) Camphenol	C_10_H_16_O	152.23	6.71
9	19.54	(H) 2(10)-pinen-3-one	C_10_H_14_O	150.22	4.19
10	20.43	2-(2,2,3-Trimethylcyclopent-3-en-1-yl) acetaldehyde	C_10_H_16_O	152.23	4.89
11	20.93	Pinocarveol	C_10_H_16_O	152.23	7.65
12	22.07	(H) 3-Cyclohexen-1-ol	C_7_H_12_O	112.17	13.91
13	22.20	(R) Benzenemethanol	C_7_H_8_O	108.14	6.08
14	22.44	(R) 3-Cyclohexen-1-methanol	C_7_H_12_O	112.17	9.71
15	22.69	Myrtenal	C_10_H_14_O	150.22	13.81
16	23.08	3-Cyclohexen-1-ol,5-methylene-6-(1-methylethenyl)-acetate	C_12_H_16_O_2_	192.25	2.53
17	24.11	Carvone	C_10_H_14_O	150.22	5.94
18	25.25	Phellandral	C_10_H_16_O	152.23	3.15
19	25.55	Isobornyl acetate	C_12_H_20_O_2_	196.29	9.26
20	28.00	Neryl acetate	C_12_H_20_O_2_	196.29	2.86
21	28.12	Carvyl acetate	C_12_H_18_O_2_	194.27	17.77
22	28.71	Geranyl acetate	C_12_H_20_O_2_	196.29	3.11
23	29.01	(R) Copaene	C_10_H_16_	136.23	5.21
24	29.35	Cyclohexane	C_6_H_12_	84.16	14.67
25	29.74	Dodecanal	C_10_H_24_O	184.32	7.93
26	31.50	Nerylacetone	C_13_H_22_O	194.31	6.16
27	33.00	1,2,4a,5,6,8a-Hexahydro-1-isopropyl-4,7-dimethylnaphthalene	C_15_H_24_	204.35	25.49
28	33.18	*β*-Ionone	C_13_H_20_O	192.30	3.89
29	33.58	Bicyclo [4.4.0] dec-2-ene-4-ol,2-methyl-9-(prop-1-en-3-ol-2-yl)-6-[1-(hydroxymethyl)vinyl]-4,8a-dimethyl-1,2,4a,5,6,7,8,8a-octahydro-2-naphthalenol	C_15_H_24_O_2_	236.35	5.16
30	33.96	1,2,4a,5,6,8a-Hexahydro-1-isopropyl-4,7-dimethylnaphthalene	C_15_H_24_	204.35	4.56
31	34.14	1-Methyl-4-(6-methylhepta-1,5-dien-2-yl) cyclohex-1-ene	C_15_H_24_	204.35	4.40
32	34.36	1-Methyl-4-(1,2,2-trimethylcyclopentyl) benzene	C_15_H_22_	202.33	10.72
33	34.65	4-Isopropenyl-4,7-dimethyl-1-oxaspiro[2.5]octane	C_12_H_20_O	180.29	3.83
34	34.96	1,6-Dimethyl-4-isopropyltetralin	C_15_H_22_	202.33	6.20
35	35.89	(H) *π* calacorene	C_15_H_22_	200.32	2.81
36	36.43	Caryophyllene epoxide	C_15_H_24_O	220.35	22.88
37	37.38	*β*-Spathulenol	C_15_H_24_O	220.35	5.27
38	37.56	Tricyclo [5.2.2.0(1,6)]undecan-3-ol, 2-methylene-6,8,8-trimethyl-2-methylene-6,8,8-trimethyl-tricyclo[5.2.2.0(1,6)]undecan-3-ol	C_15_H_24_O	220.35	88.77
39	37.76	Isoaromadendrene epoxide	C_15_H_24_O	220.35	17.44
40	37.88	6-methyl-3-[(2Z)-6-methylhepta-2,5-dien-2-yl]-7-oxabicyclo[4.1.0]heptane	C_15_H_24_O	220.35	100
41	38.30	Jasmoline I	C_21_H_30_O_3_	330.50	18.61
42	39.03	Schembl21824483	C_15_H_24_O	220.35	24.81
43	39.58	1-Oxaspiro[2.5]octane,5,5-dimethyl-4-(3-methyl-1,3-butadienyl)-5,5-dimethyl-4-[(1E)-3-methyl-1,3-butadienyl]-1-oxaspiro[2.5] octane	C_14_H_22_O	206.32	11.21
44	39.68	Patchoulane	C_15_H_26_	206.37	6.35
45	39.99	(R) Caryophyllene	C_15_H_24_	204.35	6.89
46	40.18	3,7,11-Trimethyldodeca-6,10-dien-1-yn-3-ol	C_15_H_24_O	220.35	5.31
47	40.72	Aromadendrene oxide-(2)	C_15_H_24_O	220.35	8.85
48	40.93	(H) Caryophyllene oxide	C_15_H_24_O	220.35	14.58
49	41.03	(7S,9As)-4,4,7,9a-Tetramethyl-1,2,3,6,8,9-hexahydrobenzo [7] annulen-7-ol	C_15_H_26_O	222.37	20.96
50	41.23	1,2-Bis(ethenyl)-4-propan-2-ylidenecyclohexane	C_13_H_20_	176.30	12.97
51	41.87	4a,10a-Methanophenanthren-9*β*-ol,11-syn-bromo-1,2,3,4,4a,9,10,10a-octahydro	C_18_H_24_O_2_	272.38	6.97
52	42.16	Diepicedrene-1-oxide	C_15_H_24_O	220.35	11.10
53	42.44	7R,8R-8-Hydroxy-4-isopropylidene-7-methylbicyclo [5.3.1] undec-1-ene	C_15_H_24_O	220.35	27.61
54	43.27	(H) 10,12-Tricosadiynoic acid	C_23_H_38_O_2_	364.50	9.58
55	43.46	(H) Cubenol	C_15_H_26_O	222.37	6.40
56	44.98	4,6,6-Trimethyl-2-[(1E)-3-methylbuta-1,3-dienyl]-3-oxatricyclo [5.1.0.02,4] octane	C_15_H_22_O	218.33	8.16
57	46.48	(H) Andrographolide	C_20_H_30_O_5_	350.40	7.45
58	49.34	6,10,14-Trimethylpentadecan-2-one	C_18_H_36_O	268.50	29.74
59	49.45	Boronal	C_14_H_22_O	206.32	5.41
60	52.84	Farnesylacetone	C_18_H_30_O_5_	262.40	6.55
61	60.97	2(3H)-Furanone, dihydro-5-undecyl	C_15_H_28_O_2_	240.38	4.51
62	67.61	(R) Heptacosane	C_27_H_56_	380.70	2.98
63	70.89	1-(4-Bromobutyl)-2-piperidinone	C_9_H_16_BrNO	234.13	3.58

## Data Availability

The data used to support the findings of this study are included in the article. Further data or information required are available from the corresponding author upon request.

## Abbreviations

KA: Kainic acid
GC-MS: Gas chromatography-mass spectrometry
GABA: *γ*-Aminobutyric acid type a
COX2: Cyclooxygenase2
TLR4: Toll-like receptor 4
HMBG1: High mobility group box 1
GAD: Glutamic acid decarboxylase
GFAP: Glial fibrillary acidic protein
GIRK: G protein gated inwardly rectifying K channels
NMDAR: N-methyl-D-aspartic acid receptor
MAP: Microtubule-associated protein
TLE: Temporal lobe epilepsy
AEDs: Antiepileptic drugs
TCM: Traditional Chinese medicine
FID: Flame ionization detection
CCK-8: Cell counting kit-8
DAB: 3,3'-Diaminobenzidine tetrahydrochloride
HE: Hematoxylin-eosin
ELISA: Enzyme-linked immunosorbent assay
BAO: The aerial parts of *B. chinense* aromatic oil.
