# Supplementary material for: The Aerial Parts of Bupleurum Chinense DC. Aromatic Oil Attenuate Kainic Acid-Induced Epilepsy-Like Behavior and Its Potential Mechanisms
Source: Biomed Res Int. 2022 Apr 11;2022:1234612. doi: 10.1155/2022/1234612 (PMC9015862; doi:10.1155/2022/1234612)

**Supplemental Files**

Figure S1 The GC chromatogram of BAO.


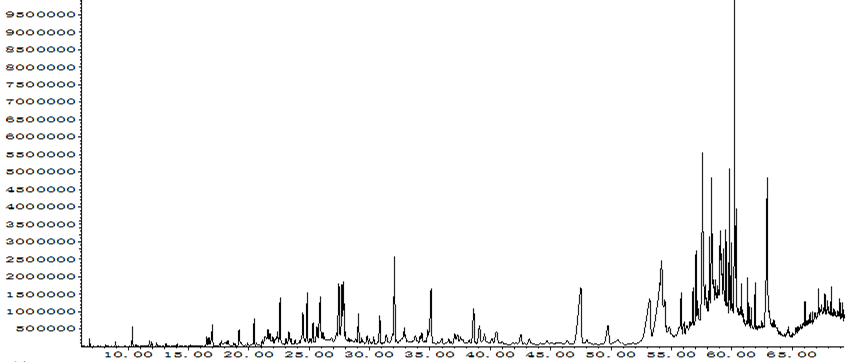


Figure S2 The MS of all major identified components.


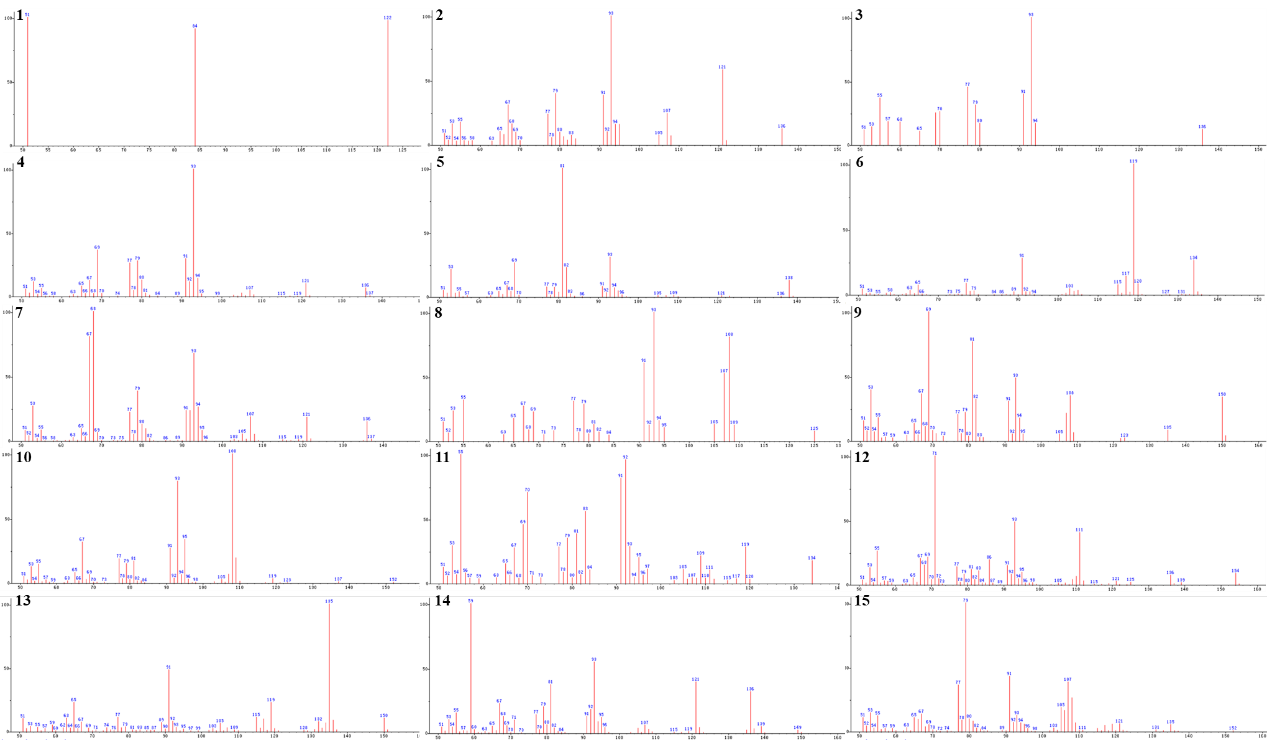


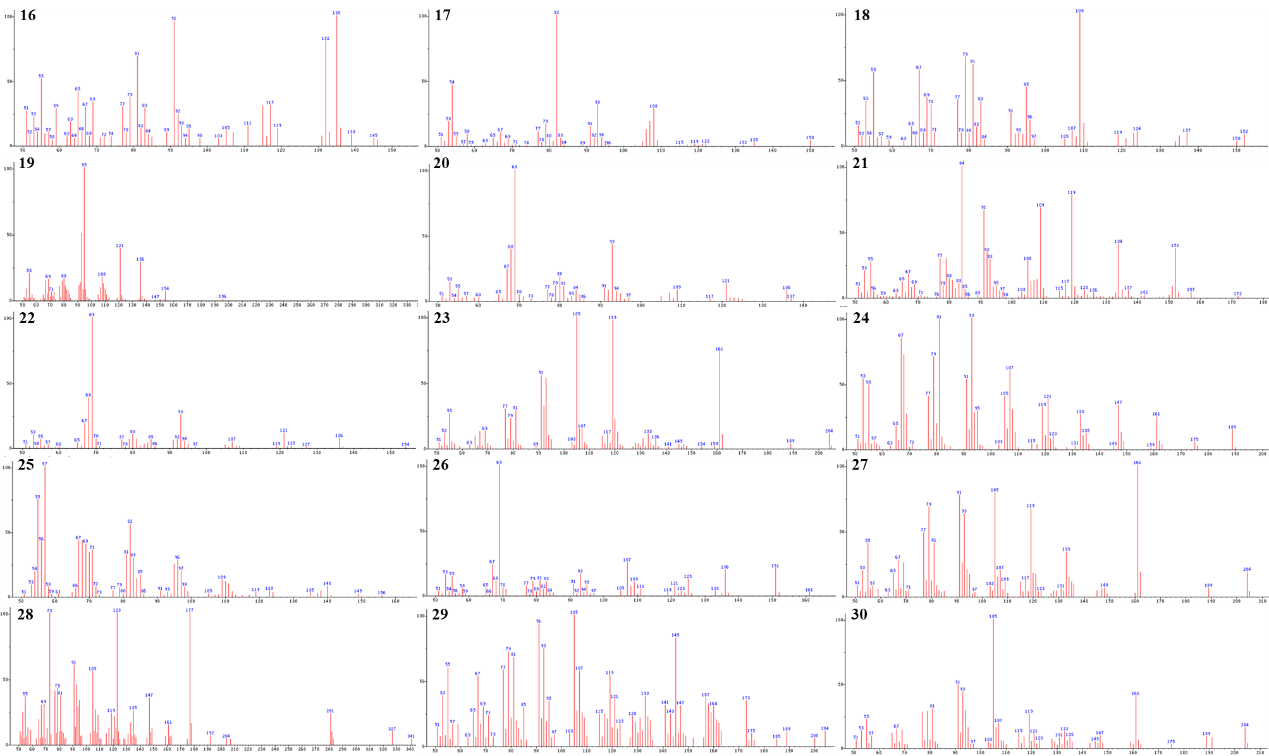


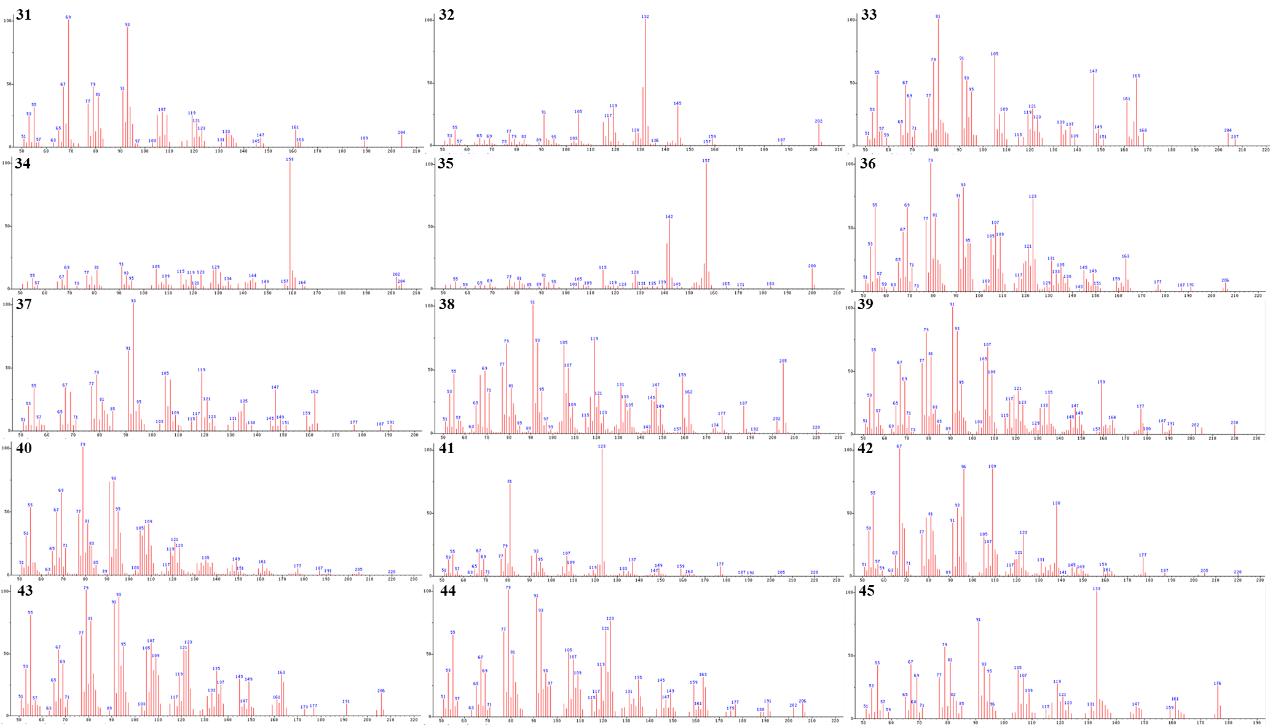


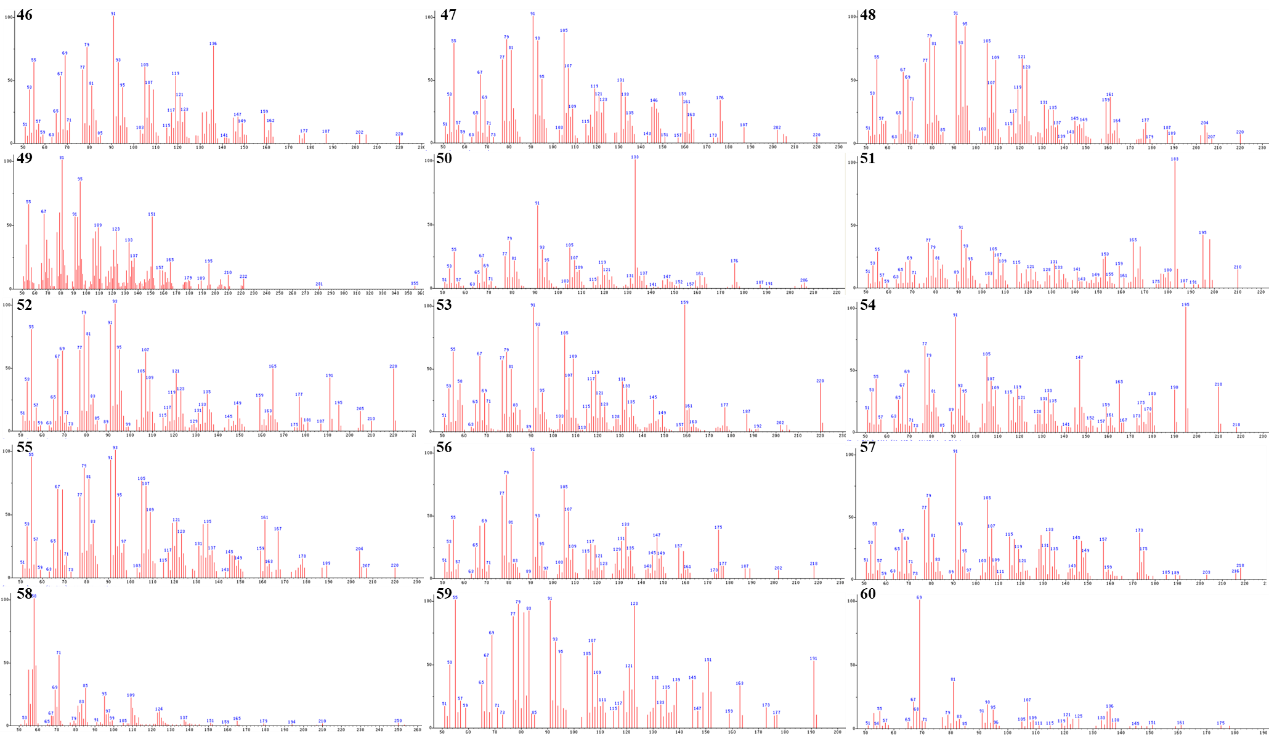


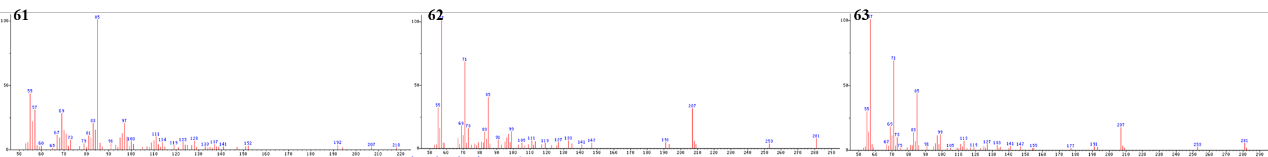

Supplement: Supplementary Materials — Supplementary data associated with this article can be found in the Supporting Information. [file 1234612.f1.docx]
